# Acetazolamide Tolerance in Acute Decompensated Heart Failure: An Observational Study

**DOI:** 10.3390/jcm13123421

**Published:** 2024-06-11

**Authors:** Ignacio Sosa Mercado, Sophie Putot, Elena Fertu, Alain Putot

**Affiliations:** 1 Department of Cardiology and Vascular Diseases, Hôpitaux du Pays du Mont Blanc, 74700 Sallanches, France; i.sosa-mercado@ch-sallanches-chamonix.fr (I.S.M.); s.putot@ch-sallanches-chamonix.fr (S.P.); e.fertu@ch-sallanches-chamonix.fr (E.F.); 2 Department of Internal Medicine and Infectious Diseases, Hôpitaux du Pays du Mont Blanc, 74700 Sallanches, France

**Keywords:** heart failure, diuretics, acetazolamide, safety, tolerance, side effects

## Abstract

**Objectives:** This real-life study aimed to evaluate the safety of acetazolamide (ACZ), a carbonic anhydrase inhibitor with diuretic effects. ACZ has recently been proven to improve decongestion in the context of patients hospitalized for acute heart failure (HF). However, data in terms of safety are lacking. **Methods:** We conducted a monocentric observational prospective study from November 2023 to February 2024 in a 12-bed cardiology department, recording adverse events (hypotension, severe metabolic acidosis, severe hypokalemia and renal events) during in-hospital HF treatment. All patients hospitalized for acute HF during the study period treated with ACZ (500 mg IV daily for 3 days) on top of IV furosemide (*n* = 28, 48.3%) were compared with patients who have been treated with IV furosemide alone (*n* = 30, 51.7%). **Results:** The patients treated with ACZ were younger than those without (median age 78 (range 67–86) vs. 85 (79–90) years, respectively, *p* = 0.01) and had less frequent chronic kidney disease (median estimated glomerular fraction rate (60 (35–65) vs. 38 (26–63) mL/min, *p* = 0.02). As concerned adverse events during HF treatment, there were no differences in the occurrences of hypotension (three patients [10.7%] in the ACZ group vs. four [13.3%], *p* = 0.8), renal events (four patients [14.3%] in the ACZ group vs. five [16.7%], *p* = 1) and severe hypokalemia (two [7.1%] in the ACZ group vs. three [10%], *p* = 1). No severe metabolic acidosis occurred in either group. **Conclusions:** Although the clinical characteristics differed at baseline, with younger age and better renal function in patients receiving ACZ, the tolerance profile did not significantly differ from patients receiving furosemide alone. Additional observational data are needed to further assess the safety of ACZ–furosemide combination in the in-hospital management of HF, especially in older, frail populations.

## 1. Introduction

Current guidelines recommend a loop diuretics-based treatment to achieve euvolemia and decongestion for all patients with acute heart failure (HF) presenting signs and symptoms of volume overload [1]. A position paper from the HF Association of the European Society of Cardiology refers to this setting and discusses the practical use of diuretics, suggesting different strategies according to the clinical response [2]. Despite these recommendations, the persistence of signs of congestion at discharge still happens and has been shown to be an important prognostic variable [3,4]. Acetazolamide (ACZ) is a carbonic anhydrase inhibitor which blocks sodium bicarbonate reabsorption in the proximal tubules, and is a forgotten diuretic previously used in HF [5,6]. The addition of ACZ (500 mg intravenous (IV) bolus) on top of loop diuretics has been shown to improve the diuretic effect, confirmed by increasing natriuresis per dose of furosemide equivalent, which is an excellent indicator of efficacy [6,7]. Additionally, ACZ has an intrinsic renal vasodilatory effect, protecting the nephrons against ischemia-reperfusion damage [8]. Another interesting characteristic of ACZ is its power to deliver chloride to the macula densa cells, decreasing renin production and reducing neurohumoral activation [5].

The recent multicentre, double-blind, randomized, placebo-controlled ADVOR trial (ACZ in Acute Decompensated Heart Failure with Volume Overload), which enrolled 519 patients with acute decompensated HF with clinical signs of volume overload and a B-type natriuretic peptide (BNP) of >250 pg/mL or an N-terminal proBNP (NT-proBNP) of >1000 pg/mL, has compared the efficacy of decongestion with ACZ versus placebo. Patients were randomized to receive an IV bolus of ACZ 500 mg once daily for 3 days or placebo on top of standardized IV loop diuretics. The primary endpoint of successful decongestion, defined as the absence of congestive signs within the 3 days after randomization without escalation of decongestive therapy, was observed in 108 patients (42.2%) in the ACZ group versus (vs.) 72 patients (30.5%) in the placebo group (risk ratio 1.46, 95% CI 1.17–1.82; *p* < 0.001), and the secondary endpoint of length of hospital stay was about 1 day shorter with ACZ (8.8 [95% CI 8.0–9.5] vs. 9.9 [95% CI 9.1–10.8] days). No significant differences were found for other outcomes or adverse events like severe metabolic acidosis, renal events, severe hypokalemia and hypotension [9].

During the 2023 Congress of the European Society of Cardiology, a focused update of the 2021 Guidelines for the Diagnosis and Treatment of Acute and Chronic HF was published [1]. The task force summarized the results of the ADVOR trial and suggested that ACZ on top of loop diuretics may be helpful for achieving successful decongestion in acute HF settings [1,9]. Nevertheless, further data on safety and security are needed [1]. These recommendations were quickly implemented in our cardiology department. We report here the observational data from the first months following the implementation of this new protocol.

A pre-specified analysis of the ADVOR trial on kidney injury shows that ACZ was associated with a higher incidence of worsening renal function (WRF—rise in creatinine ≥ 0.3 mg/dL) during the treatment period (40.5% vs. 18.9%; *p* < 0.001), but there was no difference in creatinine after 3 months. This WRF during hospitalization was not associated with a higher incidence of HF hospitalizations or mortality and did not modify the treatment effect of ACZ on the combined endpoint. Moreover, proportionally higher natriuresis and diuresis were observed in patients with lower renal function [9,10]. Another study demonstrated that low serum chloride is strongly associated with poor diuretic response and worsening heart function [11].

However, while interventional study data are encouraging for the safety of ACZ, further observational data are needed on the real-life tolerability of this suggested combination. Indeed, patients in clinical practice differ greatly from those included in trials due to their older age and more frequent comorbidities, which substantially increase the risk of treatment-related adverse events. In the present real-life study of patients hospitalized for HF in our cardiologic ward, we sought to evaluate whether the adjuvant therapy of ACZ with furosemide was associated with a higher incidence of adverse events (renal events, hypotension, hypokalemia and severe metabolic acidosis) compared with loop diuretics alone.

## 2. Methods

### 2.1. Study Design

This monocentric, prospective observational study was conducted from September 2023 to February 2024, in the 12-bed cardiology department of Sallanches Hospital, France.

Each participant agreed to participate before inclusion. This study was conducted using data collected for clinical purposes, all of which were made anonymous in accordance with the requirements of the local ethics committee (*Comité de Protection des Personnes*) and with the Declaration of Helsinki.

### 2.2. Population

The population consisted of all consecutive patients hospitalized for acute HF in the cardiology department during the study period and receiving IV furosemide treatment during hospitalization. There were no exclusion criteria.

We compared patients treated with ACZ + furosemide with patients treated with furosemide alone.

All patients were treated with IV furosemide in the two groups, at doses at their clinicians’ discretion. Patients in the ACZ group received 500 mg IV ACZ once daily for 3 days on top of IV furosemide [9].

### 2.3. Collected Data

For each enrolled subject, demographic characteristics, including gender and age, were collected, as well as comorbidities and cardiovascular history (diabetes, dyslipidemia, atrial fibrillation, stroke, hypertension, peripheral artery disease, coronary artery disease, chronic bronchitis, cirrhosis, neurocognitive disorders and active neoplasia).

Cardiovascular co-prescriptions for HF, i.e., beta-blockers, angiotensin-converting enzyme inhibitors, angiotensin receptor blockers, angiotensin receptor-neprilysin inhibitor, sodium-glucose co-transporter 2 inhibitors (SGLT2i) and other diuretic treatments like hydrochlorothiazide were collected, as well as oral furosemide doses. The proportion of patients receiving optimal HF medical treatment, defined as the guideline-directed medical therapy (GDMT) recommended by the European Society of Cardiology [1], was reported for each group.

The histories of implanted devices as part of treatment of HF, i.e., cardiac resynchronization therapy and implantable cardioverter-defibrillator were also recorded.

The clinical data recorded at admission were systolic blood pressure (SBP), body weight, presence of pleural effusion assessed by echography or radiography, New York Heart Association functional class, lower limb oedema and left ventricular ejection fraction (LVEF).

Chloride, sodium, potassium, bicarbonate, urea, creatinine, BNP plasmatic rates and estimated glomerular filtration rates (eGFRs) (according to the CKD-EPI formula, expressed as mL/min/1.73 m^2^) were also collected at admission.

In-hospital clinical data collected were daily SBP, loss of weight, first 24 h diuresis, need for iron infusion and amines, dose of IV furosemide in the first 24 h and maximal IV furosemide dose. The in-hospital evolution of biological data (chloride, sodium, potassium, bicarbonate, urea, creatinine plasmatic rates and eGFR) with nadir values and at discharge were recorded.

Reported adverse events included the presence of hypotension (SBP < 85 mmHg), severe metabolic acidosis (HCO_3_^−^ ≤ 12 mmol/L), severe hypokalemia (K^+^ < 3 mmol/L) and renal events (doubling of baseline serum creatinine level, decrease of ≥50% baseline eGFR or need for dialysis) [9].

### 2.4. Statistical Analysis

The two groups were compared for all recorded data. Given the limited number of patients and the non-Gaussian distribution, continuous variables were expressed as medians and interquartile ranges (IQR) and categorical variables as numbers and percentages. Continuous variables were compared using the nonparametric Mann–Whitney U-test, and categorical variables using the Chi-squared and Fisher’s tests, when appropriate. Statistical significance was defined as *p* < 0.05.

As this exploratory study did not attempt to employ a hypothetico-deductive approach, the number of patients included was not pre-defined but rather corresponded to a specific study period. Given the small number of patients and events, no multivariate analysis was performed. This prospective study, based on clinical and biological data systematically collected as part of the service protocol, did not contain any missing data.

## 3. Results

### 3.1. Baseline Characteristics

A total of 58 patients were included during the study period. Among these patients, 28 (48.3%) received ACZ and 30 (51.7%) did not. Patients with ACZ were significantly younger than those without (median age 78 (range 67–86) vs. 85 (79–90) years, respectively, *p* = 0.01) and more frequently male (64.3% vs. 40%, *p* = 0.06). Preserved LVEF accounted for 60% in both groups. Chronic kidney disease (CKD) was frequent at admission in both groups, although the eGFR was significantly higher in the ACZ group (60 (35–65) vs. 38 (26–63) mL/min, *p* = 0.02) and the creatinine level rate was consequently lower (91 (76–125) vs. 120 (83–163) micromol/L, *p* = 0.06). Other comorbidities tended to be less frequent in the ACZ group (diabetes 28.6 vs. 36.7%, *p* = 0.5; hypertension 70.0 vs. 53.6%, *p* = 0.2). Fewer patients who received ACZ had histories of AF (39.3% vs. 66.7%, *p* = 0.04) and consequently less frequently received beta-blockers, while the frequency of other HF treatments did not significantly differ. Optimal HF treatment was as rare in both groups (10.7% vs. 6.7%, *p* = 0.7). Moreover, the baseline dose of furosemide was significantly lower in the ACZ group (0 (0–30) vs. 20 (0–80) mg, *p* = 0.01).

Other baseline treatments and characteristics were similarly distributed between the two groups (Table 1).

### 3.2. Management

The initial dose of IV furosemide was significantly lower in the ACZ group (80 (80–120)) mg than in the non-ACZ group (125 (110–145) mg, *p* = 0.004), as was the maximal dose (122 (80–125) vs. 125 (120–250) mg, *p* = 0.04).

However, diuresis in the first 24 h did not significantly differ between the two groups (2.3 L (1.5–2.7) in the ACZ group vs. 1.8 L (1.3–2.5) in the non-ACZ group, *p* = 0.5) and consequently neither did weight loss (4 (3–8) vs. 3 (2–5) kg, *p* = 0.2).

There was no difference in the need for iron infusion and none of the patients required amines.

Optimal HF GDMT at discharge was more frequent in the ACZ group (15 (55.6%) vs. nine (30%), *p* = 0.05, Table 2).

### 3.3. Biological Results

**Figure 1:**
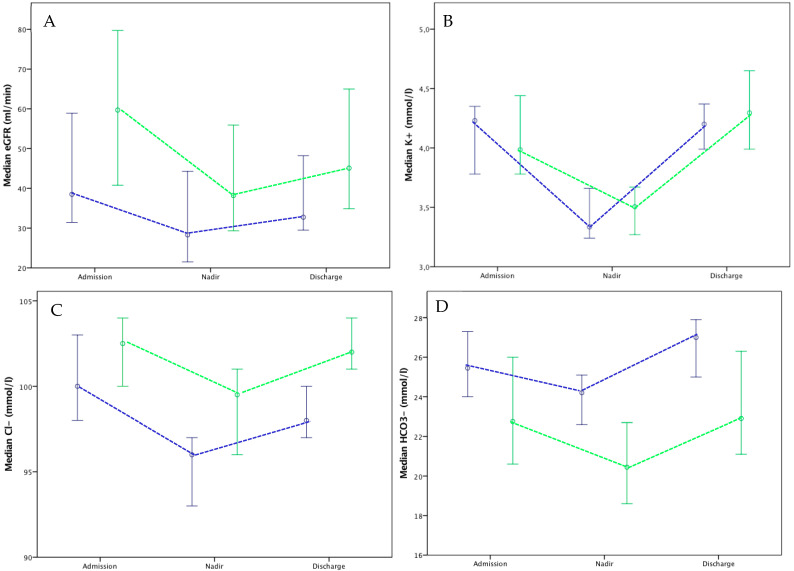
Evolution of the median (95% confidence interval) estimated glomerular fraction rate (eGFR) (**A**), potassium (**B**), chloride (**C**) and bicarbonate (**D**) plasmatic rates in patients treated by acetazolamide and furosemide (green) versus furosemide alone (blue) for acute heart failure.

Concerning biological results (Figure 1), chloride rates were higher in patients treated with ACZ than in those without, as shown by the highest values in-hospital (99 (95–101) vs. 96 (92–98) mmol/L, respectively, *p* = 0.008) and at discharge (102 (99–104) vs. 98 (96–102) mmol/L, *p* = 0.005). The bicarbonate rate was significantly lower in the ACZ group for the nadir value (20 (18–23) vs. 24 (22–27) mmol/L, *p* < 0.001) and at discharge (23 (20–27) vs. 27 (23–28) mmol/L, *p* = 0.002). No differences in urea, potassium and sodium rates were observed. The eGFR was higher in the ACZ group for the in-hospital nadir (38 (28–60) vs. 28 (18–47) mL/min/1.73 m^2^, *p* = 0.05) and at discharge (45 (33–70) vs. 33 (26–55) mL/min/1.73 m^2^, *p* = 0.07). BNP for the two groups at discharge did not significantly differ (259 (155–443) vs. 352 (254–667) ng/L, *p* = 0.3).

### 3.4. Adverse Events

No differences were noted for the occurrences of hypotension (three patients [10.7%] in the ACZ group vs. four [13.3%], *p* = 0.8), renal events (four patients [14.3%] in the ACZ group vs. five [16.7%], *p* = 1) and severe hypokalemia (two [7.1%] in the ACZ group vs. three [10%], *p* = 1). Severe metabolic acidosis did not happen in either of groups.

### 3.5. In-Hospital Events

In-hospital events (i.e., cardiogenic shock, respiratory tract infection, new onset of atrial fibrillation and in-hospital death) were as frequent in the two groups. Renal events occurred in 14.3% vs. 16.7% of patients with and without ACZ, respectively (*p* = 1).

Weight did not significantly differ at discharge between the two groups. No difference in hospitalization length was observed (median hospital stay: 7.5 days).

One-month unscheduled rehospitalization was less frequent in patients receiving ACZ compared with patients who did not (0 vs. five [16.7%], *p* = 0.05).

## 4. Discussion

In this study, we investigated the risk of adverse events using the carbonic anhydrase inhibitor ACZ in addition to a loop diuretic (furosemide) for patients with acute HF and signs of congestion. The background literature supports its use in this context, but safety data are still lacking [1,2,9,10,12].

Several combined diuretic strategies have been proposed for HF treatment [13,14]. Among them, hydrochlorothiazide has been shown to be associated with a higher incidence of worsening renal failure and hypokalemia compared with ACZ combinations or loop diuretics alone [15,16,17]. Mineralocorticoid receptor antagonists are already part of the recommended medical therapy for HF with reduced LVEF [1].

Using the same definitions for adverse events as the ADVOR trial, we aimed to compare the safety of ACZ + furosemide versus furosemide alone in a “real-world” situation of predominantly frail old patients hospitalized for acute HF, mostly with preserved LVEF. Although the limited number of patients and the observational design does not allow us to definitively conclude the absence of a difference between the two groups, these “real world” data confirm a good safety profile, with no severe metabolic acidosis and a similar distribution of adverse events (hypokalemia, acute renal failure and low blood pressure).

Similarly, data extracted from the ADVOR trial show that the incidences of adverse events, including hypokalemia, renal events and hypotension, were similar in the ACZ group and placebo group (3.1% versus 1.2%, respectively; *p* = 0.14) and no severe metabolic acidosis was observed [9,18], as in the present study. Although patients treated with ACZ showed a modest decrease in mean potassium levels during the treatment period, the incidences of hypokalemia (<3.5 mmol/L) did not significantly differ from patients without ACZ. Severe hypokalemia occurred in only 1% overall, and the rates were similar in both groups [9]. These percentages were lower than in our study, where the rate of severe hypokalemia was about 8.6% (two patients with ACZ and three without (*p* = 1)), reflecting ‘real life’ conditions with frail older patients. According to the DIURESIS-HF randomized trial (Diamox/Aldactone to Increase the Urinary Excretion of Sodium: an Investigational Study in Congestive HF), which investigated the diuretic efficiency of ACZ combined with loop diuretic therapy in 34 patients with acute HF at high risk of diuretic resistance, no difference for the decrease in serum potassium levels was found between the high-dose loop diuretic group and the combinational treatment with ACZ group [19]. However, on the other hand, the CLOROTIC trial (Combining Loop with Thiazide Diuretics for Decompensated HF), which also assessed the impact of decongestion combinational strategy versus loop diuretic-only strategy, highlighted a higher incidence of severe hypokalemia in patients treated with hydrochlorothiazide (40.6% vs. 16.1%, *p* = (0.001)) [16].

The pre-specified analysis of the ADVOR trial assessing renal function and decongestion with ACZ found that ACZ had a pronounced effect on natriuresis and diuresis in patients with lower eGFR [10]. However, despite a trend in higher diuresis under ACZ-furosemide association than under furosemide alone, we found no significant difference between the two groups for the first 24 h of diuresis and loss of body weight. In the ADVOR trial, the authors found that WRF during the treatment period was twice as frequent with ACZ as without (40.5% vs. 18.9%; *p* < 0.001). This WRF during hospitalization was not associated with a higher incidence of HF hospitalizations or mortality [10]. Similarly, in the CLOROTIC trial, an increase in serum creatinine occurred more frequently in patients on hydrochlorothiazide than placebo (46.5% vs. 17.2%, respectively, *p* < 0.001) [1,16,17]. Some authors suggest that ACZ in combination with loop diuretics could be a safer strategy than thiazide and loop diuretics in association in terms of WRF and hypokalemia risks [1,17]. Noteworthy, the reduction in blood pressure during acute decompensated HF is strongly associated with WRF [20].

In the present study, patients with ACZ-furosemide association had a higher median eGFR before treatment than patients with furosemide alone, in part because ACZ is contra-indicated in cases of severe renal failure and also because practitioners were probably more reluctant to introduce ACZ for the most fragile patients. Thus, comparison of eGFR evolution under treatment is limited. However, as shown in Figure 1, evolution curves of median eGFR during hospital stays were similar in the two groups. Renal failure has a major prognostic value in cardiology settings, not only for predicting the progression of kidney disease but also cardiovascular outcomes [21].

Many studies have demonstrated the prognostic significance of hypochloremia in patients with HF, regardless of the LVEF [22,23,24]. Interestingly, the lower chloride plasmatic rates observed at discharge in the group without ACZ could be interpreted as worse electrolyte balance and a predictor of suboptimal response to decongestion therapy, as shown in a sub-study of the PROTECT trial and exposed by Kataoka [11,25]. In the PROTECT trial, low baseline chloride was associated with high bicarbonate, diuretic resistance and worsening heart failure, and new or persistent hypochloremia (<96 mEq/L) was associated with increased mortality (HR 3.11 (2.17–4.46), *p* = 0.001) [11]. In 61 patients admitted for acute HF randomized to receive either 250 mg oral ACZ for 2 days or no ACZ on top of IV furosemide, Kosiorek et al. found a significant increase in serum chloride levels in the ACZ group, as compared with the control group, on day 2 and on day 3 of treatment. The difference between the two groups was not significant at baseline or at discharge [26].

Rehospitalization for HF during a 3 month follow-up period did not significantly differ between the two groups in the ADVOR trial (17.4% with ACZ vs. 18.4% without; (HR, 1.07 (0.71–1.59)) [9]. In the present study, unscheduled rehospitalization within one month was less common in patients treated with ACZ. However, this probably related more to the different baseline characteristics of the two groups (patients without AZT were frailer) than to the treatment itself. Persistent congestion and excessive decongestion can lead to urgent consultation or rehospitalization. These two situations are more common in older patients with impaired renal function, due to the low blood pressure side effect and diuretic resistance [15,16].

Patients with ACZ were more likely to have a lower dose of furosemide during the in-hospital period. Because of WRF-related diuretic resistance in patients without ACZ, higher doses of furosemide may be required to achieve the same diuretic response. Sodium reabsorption in proximal tubules is increased in patients with diuretic resistance, resulting in weak effectiveness of loop diuretics [4,5,7,27]. Of particular interest is the use of drugs that act at a different site of action in the nephron by blocking sodium reabsorption in the proximal tubule, offering more sodium to the loop of Henle, thereby reinforcing the effect of loop diuretics [19,28]. This diuretic effect could also be enhanced by the addition of other pro-natriuretic drugs such as SGLT2i, which were only rarely prescribed at admission in our study. Despite encouraging security and efficacy data in older patients, previous reports have already highlighted low SGLT2i prescription rates in this population [29].

Finally, optimal HF therapy at discharge was more often observed in the ACZ group, which could be due to optimized medical treatment in these patients exposed to a new pharmacology strategy, as well as to the fact that the patients were older and more comorbid in the other group.

### Limitations

Several limitations have to be acknowledged. First, as for all observational studies, the main limitation was the difference at baseline between the two groups. Older and worse renal function patients were more frequently present in the group without ACZ, making it difficult to compare the evolution of renal parameters between the two groups. Moreover, the limited number of patients and the low incidence of events prevented the use of multivariate analyses to account for confounding factors. These differences between the two groups on admission, and the lack of adjustment for the main confounding factors (age, renal function and comorbidities), limit the interpretability of the results; given their lower frailty, patients on ACZ were less likely to present adverse events, regardless of treatment tolerance. Second, it is likely that the study was underpowered to find a difference in the incidence of infrequent events. However, we believe that this “real-life” study carried out in a conventional cardiology unit illustrates the safety and ease of use of ACZ in everyday practice, independently of its effectiveness, which was not intended to be assessed in this study. Indeed, the previous ADVOR study has successfully proved the primary endpoint of decongestion [9]. Only a prospective interventional study with a trained team and a larger number of patients can assess these kinds of outcomes.

## 5. Conclusions

In this observational study, we investigated the safety and tolerance of the association of ACZ with furosemide recently suggested by the ESC, compared with standard of care (furosemide alone) for in-hospital treatment of HF. Although the clinical characteristics differed at baseline, with younger and less comorbid ACZ patients, the tolerance profiles appeared similar in the two groups. However, given the limitations of small numbers and a monocentric retrospective design, we cannot exclude a higher risk of rare adverse events resulting from the proposed strategy. Moreover, an enhanced diuretic strategy appears to be less likely to be prescribed in older multi-morbid patients. Additional observational data are needed to further assess the tolerability of this diuretic regimen in this high-risk population, increasingly frequently in hospital. Before extending this combination therapy to older patients hospitalized for decompensated HF, the benefit–risk balance of this treatment has to be specifically evaluated in large-scale hospital cohorts, optimally collecting data not only on safety and effectiveness, but also on functional status and quality of life.

## Figures and Tables

**Table 1:** Patient characteristics on admission (*n* (%) or median (IQR)).

	With Acetazolamide (*n* = 28)	Without Acetazolamide (*n* = 30)	*p*
Demographics/comorbidities			
Age (years)	78 (67–86)	85 (79–90)	0.01
Male	18 (64.3)	12 (40.0)	0.06
Chronic bronchitis	6 (21.4)	6 (20.0)	0.9
Dyslipidemia	13 (46.4)	22 (73.3)	0.04
Cirrhosis	0	1 (3.3)	1
Dementia	1 (3.6)	5 (16.7)	0.2
Active neoplasia	3 (10.7)	0	0.1
Cardiovascular history			
Stroke	3 (10.7)	7 (23.3)	0.3
Hypertension	15 (53.6)	21 (70.0)	0.2
Atrial fibrillation	11 (39.3)	20 (66.7)	0.04
Peripheral artery disease	3 (10.7)	8 (26.7)	0.1
Coronary artery disease	7 (25.0)	11 (36.7)	0.4
Diabetes	8 (28.6)	11 (36.7)	0.5
Stage 2 CKD	10 (35.7)	8 (26.6)	0.2
Stage 3 CKD	11 (39.2%)	13 (43.3%)	
Stage 4–5 CKD	3 (10.7%)	8 (26.6%)	
Clinical presentation			
SBP (mmHg)	130 (106–145)	130 (116–152)	0.2
Weight (kg)	77 (62–84)	77 (55–92)	1
NYHA II	0	3 (10.0)	0.2
NYHA III	14 (50.0)	11 (36.7)	
NYHA IV	14 (50.0)	16 (53.3)	
Pleural effusion	15 (53.6)	20 (66.7)	0.3
Lower limb oedema	22 (78.6)	23 (76.7)	0.9
LVEF (%)	60 (35–65)	50 (40–60)	0.6
LVEF < 41%	10 (35.7)	6 (20)	0.1
LVEF 41–49%	1 (3.6)	6 (20.0)	
LVEF > 49%	17 (60.7)	18 (60.0)	
Biological data			
BNP (ng/L)	507 (186–1246)	563 (296–1106)	0.6
Na^+^ (mmol/L)	139 (137–141)	139 (137–141)	1
K^+^ (mmol/L)	4.0 (3.7–4.6)	4.2 (3.7–4.4)	0.9
Cl^−^ (mmol/L)	102 (99–105)	100 (98–104)	0.1
HCO_3_^−^ (mmol/L)	23 (20–26)	25 (23–28)	0.01
Urea (mmol/L)	8.5 (5.3–13.7)	9.6 (6.3–13.1)	0.5
Creatinine (µmol/L)	91 (76–125)	120 (83–163)	0.06
eGFR (mL/min/1.73 m^2^)	60 (39–83)	38 (26–63)	0.02
Baseline treatment			
ACEI/ARBs	8 (28.6)	6 (20.0)	0.4
MRA	2 (7.1)	5 (16.7)	0.4
Sacubitril/valsartan	1 (3.6)	2 (6.7)	1
SGLT2i	5 (17.9)	4 (13.3)	0.6
betablockers	13 (46.4)	22 (73.3)	0.04
HCT	3 (10.7)	1 (3.3)	0.3
Optimal HF treatment	3 (10.7)	2 (6.7)	0.7
ICD	1 (3.6)	1 (3.3)	1
CRT	0	1 (3.3)	1
IV furosemide dose (mg)	0 (0–30)	20 (0–80)	0.01
0	21 (75.0)	14 (46.7)	0.05
]0–40]	4 (14.3)	3 (10.0)	
]40–120]	3 (10.7)	9 (30.0)	
>120	0	4 (13.3)	

ACEI/ARBs—angiotensin-converting enzyme inhibitors/angiotensin receptor blockers; BNP—brain natriuretic peptide; CKD—chronic kidney disease; CRT—cardiac resynchronization therapy; eGFR—estimated glomerular filtration rate; HCT—hydrochlorothiazide; HF—heart failure; ICD—implantable cardioverter-defibrillator; IV—intravenous; LVEF—left ventricular ejection fraction; MRA—mineralocorticoid receptor antagonist; NYHA—New York Heart Association; SBP—systolic blood pressure; SGLT2i—sodium-glucose cotransporter type 2 inhibitors.

**Table 2:** In-hospital management and outcomes (*n* (%) or median (IQR)).

	With Acetazolamide (*n* = 28)	Without Acetazolamide (*n* = 30)	*p*
Clinical management			
IV Furosemide, D1 (mg)	80 (80–125)	125 (110–145)	0.004
IV Furosemide, max (mg)	122 (80–125)	125 (120–250)	0.04
Vasopressive amine	0	0	1
Iron infusion	3 (10.7)	3 (10.0)	0.9
Clinical evolution			
SBP, nadir (mmHg)	96 (90–104)	99 (90–106)	0.6
SBP < 85 mmHg	3 (10.7)	4 (13.3)	0.8
Discharge Weight (kg)	70 (58–82)	74 (51–86)	0.7
Loss of weight (kg)	4 (3–8)	3 (2–6)	0.2
Diuresis, D1 (L)	2.3 (1.5–2.7)	1.8 (1.3–2.5)	0.5
Biological evolution			
BNP discharge (ng/L)	259 (155–443)	352 (254–667)	0.3
Cl^−^ nadir (mmol/L)	99 (95–101)	96 (92–98)	0.008
Cl^−^ discharge (mmol/L)	102 (99–104)	98 (96–102)	0.005
Na^+^ nadir (mmol/L)	136 (134–137)	135 (134–138)	0.9
Na^+^ discharge (mmol/L)	138 (136–139)	137 (135–140)	1
HCO_3_^−^ nadir (mmol/L)	20 (18–23)	24 (22–27)	<0.001
HCO_3_^−^ nadir < 12 mmol/L	0	0	1
HCO_3_^−^ discharge (mmol/L)	23 (20–27)	27 (23–28)	0.002
K^+^ nadir (mmol/L)	3.5 (3.2–3.7)	3.3 (3.2–3.7)	0.7
K^+^ nadir < 3 mmol/L	2 (7.1)	3 (10)	1
K^+^ discharge (mmol/L)	4.3 (3.9–4.8)	4.2 (3.8–4.4)	0.2
Creatinine nadir (µmol/L)	133 (92–208)	152 (115–219)	0.1
Creatinine discharge (µmol/L)	118 (86–170)	132 (92–168)	0.3
Urea nadir (mmol/L)	12.8 (9.5–21.1)	14.4 (9.2–24.7)	0.4
Urea discharge (mmol/L)	11.7 (8.3–18.1)	11.9 (7.4–17.3)	1
eGFR nadir (mL/min)	38 (28–60)	28 (18–47)	0.05
eGFR discharge (mL/min)	45 (33–70)	33 (26–55)	0.07
In-hospital events			
Cardiogenic shock	1 (3.6)	0	0.5
New atrial fibrillation	1 (3.6)	2 (6.7)	1
Renal events *	4 (14.3)	5 (16.7)	1
Respiratory tract infection	4 (14.3)	3 (10.0)	0.7
Hospitalization stay (days)	7.5 (5–10)	7.5 (5–14)	0.4
In-hospital death	1 (3.6)	1 (3.3)	1
Treatment at discharge			
Optimal HF treatment at discharge	15 (55.6)	9 (30.0)	0.05
Increase of basal diuretic dose	9 (32.1)	10 (33.3)	0.9
One-month unscheduled rehospitalization	0	5 (16.7)	0.05

* Defined as the doubling of serum creatinine from baseline, a decrease in eGFR of 50% or need for dialysis. BNP—B-type natriuretic peptide; eGFR—estimated glomerular fraction rate; HF—heart failure; IV—intravenous; SBP—systolic blood pressure.

## Data Availability

The raw data supporting the conclusions of this article will be made available by the authors on request.
